# The linguistic differences in concept conveying in English and Chinese xMOOC forums

**DOI:** 10.1016/j.heliyon.2022.e12551

**Published:** 2022-12-24

**Authors:** Tai Wang, Hercy N.H. Cheng, Zhiqiang Cai

**Affiliations:** a Faculty of Artificial Intelligence in Education, Central China Normal University, Wuhan, Hubei, 430079, China; b Center for General Education, Taipei Medical University, Taipei, 110301, Taiwan; c Wisconsin Center for Education Research, School of Education, University of Wisconsin-Madison, Madison, Wisconsin, 53706, United States

**Keywords:** MOOC, Teaching assistant-student interaction, Concept conveying, Linguistic features

## Abstract

Recent studies have found that comments from teaching assistants may encourage interactions in edX-like Massive Open Online Course (xMOOC) forums. However, how concepts from these interactions are conveyed to other xMOOC participants has not received much attention. Therefore, this study focuses on a unidirectional teaching assistant-student xMOOC interaction (TS interaction), a content-related pair including one question from a student and one immediate answer from a teaching assistant. The authors particularly investigate the linguistic features (i.e., concept connectivity, concept concreteness, readability and semantic overlap) of concept conveying in TS interactions with many responses (mTS) and with few responses (fTS). In addition, a language factor (English and Chinese) is also considered. Additionally, the interaction transcripts from science lectures (SL) and political briefings (PB) were used as control groups as two opposite cases of concept conveying. At the concept level, the concept conveying in transcripts were modelled as a graph, and measured by common indicators in graph theory. At the overall level, the concept conveying in transcripts were measured by regular linguistic measuring tools. The results show that interactions with mTS and fTS demonstrate different concept conveying tendencies toward SL and PB in terms of linguistic features in both languages. The results suggest that in both languages, teaching assistants may use mixed concept-conveying strategies to stimulate more follow-up responses in xMOOC forums. These conclusions drawn from TS interactions can be even partially generalized in a larger student-student (SS) interaction dataset.

## 1 Introduction

Massive open online courses (MOOCs) provide online learners with more opportunities to learn from famous instructors. Most MOOCs are designed in the form of xMOOCs, which Waks (2016) explained as edX-like massive open online courses. Usually, xMOOCs are structured as conventional courses and offer video-based lectures, assignments and assessments. In addition, forums are usually provided in xMOOCs to simulate interactions similar to those in real classrooms. In this study, a forum refers to a message board embedded in an xMOOC website, for online interaction. Although students are not generally required to participate in discussions in xMOOCs, online interactions between students and instructors or other students are essential in fostering students' positive learning experiences and outcomes (Cho and Cho, 2016). During a semester of a xMOOC when the instructor is absent, learners may feel frustrated and have difficulty learning unfamiliar course content as a result of receiving superficial or inadequate feedback from peers (Hew, 2018). In recent studies, the contributions of instructors to xMOOC forums have been further explored. Gregori et al. (2018) found that the instructor's presence and peer-instructor interactions determined MOOC completion. Goshtasbpour et al. (2019) revealed that the major contributions of instructors in forums were social and teaching related, much more than offering a cognitive presence. Furthermore, they mainly used phatic communions to facilitate group cohesion. Another similar result in (Wise and Cui, 2018) indicated that instructor comments aimed at supporting learners in finding answers could encourage interactions among learners. Since instructors' cognitive contributions in discussions are the lowest, this status cannot perfectly relieve the aforementioned complaints from learners regarding ‘receiving superficial or inadequate feedback’. One major reason for this lack of feedback is that answering questions in forums is time-consuming for instructors who have already spent tremendous time preparing course content, while also having to deal with questions from global learners in xMOOCs (Hew and Cheung, 2014; Hew, 2016). For this reason, some xMOOCs provide teaching assistants to help the chief teachers answer students' questions. In this manuscript, therefore, we focus on the role of teaching assistants in xMOOC forums. This orientation has the following two benefits: it is usually much easier to find teaching assistant engagement than chief teacher engagement in discussions; the forthcoming results can also be illuminating to chief teachers or other types of instructors.

Before our research purpose is formulated, several technical terms are defined. A unidirectional teaching assistant-student xMOOC interaction (TS interaction) in this study is a content-related pair involving one question from a student and one immediate answer from a teaching assistant. Content-related means that the pair focuses on the course content (Wise et al., 2017); any content-irrelevant question and answer pairs (e.g., students asking why credits are missing or students and teachers simply greeting each other) are not included in the analysis (more details can be found in Section 5.5: Datasets). Immediate means that no second student responds before the teaching assistant answers the question. Counter pairs, which consist of one question from a teaching assistant and one answer from a student, are not commonly observed in xMOOCs at present and are beyond the scope of the current study.

As one type of interaction, one major function of the TS interactions defined above is to convey concepts through questions and answers (Kucer, 2014; Marshall and Vashe, 2008; Zhou et al. 2015). Concept conveying can be viewed as a form of cognitive contribution (Goshtasbpour et al., 2019), and the TS interaction can be viewed as a special piece of vicarious learning material (Chi et al., 2017; Graesser and Person, 1994). However, many TS interactions in online learning forums do not have adequate responses (Onah et al., 2014; Wong et al., 2015). In a zero-response TS interaction, it cannot be determined whether the initial TS interaction conveys a concept effectively or not. For few-response TS interactions, the responses to the initial TS interaction may help researchers infer why the initial TS interaction cannot effectively convey the concept and facilitate more discussion. Actually, in interactions with fewer responses, there may be fewer opportunities to provide comprehensive insights to readers than in interactions with more responses (Rovai, 2007). The more diverse responses a TS interaction encourages, the more opportunities the concepts conveyed by the initial TS interaction may have to be extended by other participants. This is beneficial for both repliers and observers (Anmarkrud et al., 2014; Bråten et al., 2018; Richter and Maier, 2017). In fact, the initial TS interaction with more responses forms a new piece of learning material, which can broaden the horizons of students in ‘cognitive’ and ‘discipline’ categories (Chiu and Hew, 2018; Cohen et al., 2019). As a result, regardless of the roles students play in xMOOC forums, they may learn from TS interactions as a form of vicarious learning (more details will be discussed later in Section 2: Literature review).

In addition, Gillani and Eynon (2014) found that the major interactions in xMOOC forums occur through posting. In other words, the concepts in the TS interactions studied in this manuscript are mostly conveyed by language, which can be identified by linguistic features. Therefore, the research purpose of this research is to examine how concepts are conveyed in TS interactions with more responses in terms of linguistic features. In this way, the reasons why some teaching assistants are able to effectively convey concepts may be understood. The results may also be useful for xMOOC designers, teachers, and teaching assistants who are devoted to improving the xMOOC experiences of students, especially in regularly interacting with teaching assistants, as well as simply observing the interactions of others.

## 2 Literature review

### 2.1 Vicarious learning in xMOOC forums

xMOOC forums provide students with social spaces for learning from others in addition to individual learning. However, a large proportion of the students in xMOOC forums remain silent; these students are called lurkers and tend to read posts rather than replying to posts (Onah et al., 2014; Wong et al., 2015). Loizzo and Ertmer (2016) found that even though a discussion thread may have a small number of posts, it could potentially have hundreds of ‘views’. In other words, most students assimilate knowledge by observing how others interact with each other in xMOOC forums. Such behaviors can be regarded as vicarious learning, which refers to students observing or ‘listening in’ on experts or their peers so that they can learn (Cox et al., 1999). Vicarious learning commonly occurs not only in traditional classrooms (Graesser and Person, 1994) but also in forums. Furthermore, students may learn from silently watching the interactions among teachers, teaching assistants, and other students (Chi et al., 2017).

Previous studies on vicarious learning showed that deep-level-reasoning dialogs (Craig et al., 2000; Craig et al., 2006) or higher-order-thinking conversations (Vellukunnel et al., 2017; Wang et al., 2016) were identified in teacher-student interactions. Furthermore, Craig et al. (2000) found that students in the dialogs asked significantly fewer shallow questions that required short answers than those in the monologues, suggesting that dialogs might benefit student learning. Craig et al. (2006) further confirmed that deep-level-reasoning questions might lead to higher learning gains in vicarious learning. Although these questions may activate relevant concepts and the cognitive development of students, xMOOC students do not often ask deep-level-reasoning questions (Vellukunnel et al., 2017; Wang et al., 2016). For this reason, to achieve better cognitive development, they might need help from others (e.g., clarifying concepts). Fortunately, the primary function of teaching assistants in xMOOC forums is to convey concepts to help students resolve their questions first, which may likely encourage new responses to generate more vicarious learning materials. Undoubtedly, these teaching assistants may exploit the chance to answer students’ questions by proposing deeper questions.

### 2.2 Concept conveying

Concept conveying refers to a presenter expressing concepts to an audience (Kucer, 2014; Marshall and Vashe, 2008; Zhou et al., 2015). The conveying medium can be texts, images, voices, etc. In TS interactions in xMOOC forums, concepts are conveyed by texts. Textual concept conveying is studied in the following three subfields: scientific education, educational technology and discourse processes.

In scientific education, concept conveying studies focus on how to reduce intrinsic or extraneous cognitive load (Sweller et al., 1998) by filling the gap between jargon and everyday language. One approach is to develop a replicable method for training young scientists who do not receive formal training in communicating their science to the public (Brownell et al., 2013; de Bruin and Bostrom, 2013). However, it is difficult to recruit qualified teaching assistants who can dedicate a certain amount of time to giving high-quality feedback on students’ assignments. Another approach is to develop an automatic jargon identifier that can remind science lecturers of jargon that they should adjust when interacting with nonexperts (Rakedzon et al., 2017). Nevertheless, as a source of vicarious learning in xMOOCs, the concept-level features of TS interactions have not yet been investigated.

In educational technology, there are two common methods used to capture concept-conveying features. The first is a concept map (Stewart et al., 1979), which is used to assess learning quality (Hay, 2007; Whitelock-Wainwright et al., 2020). The second is corpus analysis (Stubbs, 1996), which is used to explore students’ cognitive and linguistic development. For example, Silva and Dennick (2010) used Wmatrix2 (a linguistic program for corpus analysis) to analyze cognitive processes (e.g., questioning, explaining, and reasoning) recorded in problem-based learning discussion transcripts. However, most relevant studies (e.g., Weinerth et al., 2014; Cobb and Boulton, 2015) focus on concepts conveyed by learners instead of by teaching staff. A certain concept (including its meaning and its connections to other concepts) expressed by a speaker may differ largely from the concept perceived by the audience (Carroll, 1964), similar to the interactions in Pictionary games, in which one person doodles a picture to describe a word while another person guesses what the word is. Similarly, the concepts conveyed in the interactions between teaching assistants and learners in xMOOC forums may be problematic and should be carefully examined. Therefore, a unified benchmark is needed to measure concept conveying at different levels, e.g., the concept level and the overall level.

In discourse processes, Verhoeven and Graesser (2008) organized a special issue on cognitive discourse (e.g., problem solving tasks, route description tasks) and linguistic factors (e.g., causal relational markers, connectives or signaling phrases) in interactive knowledge construction (e.g., causal explanations, plans, logical justifications), particularly with respect to verbal information sources. Their contributions indicated that linguistic markers can be used to observe the cognitive process during concept conveying.

In the past decade, epidemic network analysis (ENA) was developed to identify and measure the patterns of association between knowledge, skills, values, habits of mind, and other elements that characterize complex thinking (Shaffer et al., 2016). A typical scenario for ENA is a multiple-turn discussion among students during a problem-solving project. ENA requires researchers to first label raw discussion text with different codes, such as data, design, attribute, collaboration, etc. Then, these codes with different values are placed on a two-dimensional plane to visualize the thinking trajectory of a certain student or student group.

Although ENA is a powerful technique to visualize the cognitive process, it does not fit our research scenario for three reasons. First, ENA requires researchers to label raw discussion text with different codes. Since our dataset covers many different disciplines, the correctness of each label cannot be guaranteed due to the shortage of objective and independent labeling techniques. Second, the typical scenario for ENA is a long discussion among students, which contains several turns. In contrast, the focus of this study is the single turn between a teaching assistant and a student at the beginning of a discussion thread. Finally, we aim analyzing at linguistic features, which are not maintained in coded labels. Therefore, we must develop our own methods to answer the research questions.

On the other hand, the linguistic features of a piece of learning material may influence concept conveying in turn. For example, Metcalfe (2011) recommended a ‘desirable difficulty’ perspective, i.e., a difficult but also deliberately presented piece of learning material can promote the transfer of the knowledge to learners' long-term memory. Fesel et al. (2018) echoed such a perspective from a different angle, i.e., individual variation. They found that word reading efficiency, vocabulary knowledge and prior knowledge predicted children's digital comprehension scores.

As noted, the studies on the discourse process involve various linguistic features, which may effectively reveal the patterns of concept conveying in xMOOC TS interactions. Therefore, linguistic features are detailed in the next subsection.

#### 2.3 Linguistic features

Schleppegrell (2001) explained linguistic features as ‘the constellation of lexical and grammatical features that characterizes particular uses of language’ (p.431). There are two families of linguistic features. One is general, and the other is study-specific. The main purpose of studies on general linguistic features, which are usually designed from common sense, is to capture as many linguistic features of a text as possible. For example, Grant and Ginther (2000) described second language writing differences by lexical features (e.g., conjuncts, hedges), grammatical structures (e.g., nouns, nominalizations, modals), clause-level features (e.g., subordination, passives), etc. The study-specific linguistic features are defined according to the study task. For example, McNamara, Crossley, and McCarthy P.M. (2010) investigated cohesion (i.e., coreference and connectives), syntactic complexity (e.g., number of words before the main verb, sentence structure overlap), the diversity of words, and the characteristics of words (e.g., frequency, concreteness, imageability) in argumentative essays to assess writing quality. The general and study-specific linguistic features do not always have a clear boundary.

In the xMOOC context, Ramesh, Goldwasser, Huang, Daumé III, and Getoor (2013) used linguistic features (subjective/objective tags of a post and positive/negative tags of a post) as well as other predictors in xMOOC forum interactions to identify passive and active student engagement. Wen et al. (2014) adopted computational linguistic models to measure xMOOC learner motivation and cognitive engagement from the text of forum posts. In their study, the linguistic markers are cognitive words annotated by LIWC (linguistic inquiry and word count) word categories, first person pronouns, positive words, apply words (e.g., ‘try’, ‘use’, ‘implement’), and need words (e.g., ‘hope’, ‘want’, ‘goal’). They also used the abstraction level of a post to measure the post writer's cognitive engagement level. Their studies show a considerable predictive power of linguistic features when compared with human subjective assessment. Nevertheless, due to their research goals, they did not treat either TS interactions or conversation transcripts as a whole piece of vicarious learning material. As a result, the connections among concepts were ignored.

Learning is about making connections (Cross, 1999). These connections are facilitated by educators that are either implicitly mapping an organization of ideas onto materials or explicitly illustrating the structure of content in instructional materials (Jonassen et al., 1993). To visualize such a structure, linguists proposed that each distinct word is a vertex, and interacting words in sentences are connected by edges that form a network (Dorogovtsev and Mendes, 2001; Wilks and Meara, 2002). Network science, also known as graph theory (Harary and Norman, 1953), is a widely used mathematical tool to study the relationships between words and concepts (Nastase et al., 2015; Siew et al., 2019).

Following the above idea, graph theory was used to describe linguistic features in this manuscript (more details are provided in Section 5: Methods). We use this approach to examine whether a linguistic feature of concept-conveying in one language is shared by another language.

### 2.4 Content-related interactions in xMOOC forums

Content-related posts are posts concentrating on the content of a course, not greeting, self-introduction, bug report, or certification issues, etc. Although xMOOC forums are perceived as a natural recorder of students' minds in “thinking aloud” activities (online discussions), statistical data shows that the proportion of content-related threads is much less than researchers expect. Cui and Wise (2015) found that only a small proportion (28%) of the students’ questions in General Discussion and Q&A forums were content-related. More surprisingly, instructors addressed only 18% of all content-related threads. Cui and Wise (2015) not only observed a set of linguistic features^1^ which showed stark differences between the starting posts in content-related and non-content-related threads, but also took the advantage of such differences to develop a linguistic model, categorizing and identifying these two kinds of threads (Wise et al., 2016; Wise et al., 2017). Their work could help researchers and MOOC instructors to filter out content-related posts efficiently.

Almatrafi and Johri (2019) systematically reviewed studies on discussion forums in MOOCs. On teaching assistant-student interactions, they found opposite effects in different reports. Zhao et al. (2017) found that instructors' participation in online discussions showed an increase in the students' chance of reading the threads, and a decrease in the negative responses from students. Khalil and Ebner (2015) found that students reported the absence of instructors as the reason for their satisfaction regarding the interaction level. On the other hand, Tomkin and Charlevoix (2014) did not find any significant impact from instructors' participation on the overall completion rate, participation rate or attitudes. Poquet, Dawson, and Dowell, N. (2017) even found that in the case of no-facilitation (i.e., no course team's participation), super-posting activity is more noticeable than in the case of facilitation. Our explanations for these inconsistent observations are: 1) instructors' answers to students' questions are usually perceived as the “final” words, which usually ends a discussion thread. Therefore, the chance of following an instructor's response becomes probably much less than the one before the instructors' response; 2) Kellogg et al. (2014) and Tawfik et al. (2017) both found that most student-student online interactions did not demonstrate high level knowledge construction (did not exceed phase 3 in interaction analysis model, i.e., the negotiation and construction phase). Therefore, though super-posting activities are more noticeable in no-instructor threads than instructor-involved threads, students are not satisfied when the instructors are absent. In other words, the students need the instructors' participation in online discussions.

Since instructors' participation in online discussion is necessary for students’ learning, and it is limited by the available time (Khalil and Ebner, 2015), can we find some alternative strategies to improve the efficiency of TS interactions, so that one TS interaction can bring more following posts than usual? Before we reach this goal, we have to explore the linguistic differences between TS interactions with more responses and fewer responses.

## 3 Problem statements

In xMOOC forums, discussion threads with more responses suggest that students are more engaged in questioning, answering, elaborating, or debating some concepts. For this reason, to examine concept conveying in xMOOC forums, TS interactions with more responses (mTS) are distinguished from those with fewer responses (fTS).

Intuitively, professionality seems a natural perspective to observe the differences between mTS and fTS interactions; i.e., if the content of a TS interaction is too professional regarding the knowledge conveyed, lurkers may not be willing to take part in the discussion thread, on the one hand. On the other hand, lurkers may also volunteer to post questions about professional knowledge details; thus, the discussion thread may still be able to attract potential participators. Therefore, the logistic consequence derived from the professionality perspective is not certain. It deserves an exploration of the characteristics of TS interactions whose replies are either many or few. Experimental results are needed to answer the question. In addition, these interactions were compared with two different forms of interactions as control groups. One is audience-scientist interactions in science lectures (SL), while the other is reporter-spokesperson interactions in political briefings (PB). Scientists and spokespersons are well trained to convey concepts in their expertise but in different ways. Ideally, scientists are apt to convey jargon to a small circle of scholars, while spokespersons are good at packaging ideas to disseminate to a massive group of ordinary people. For these reasons, SL can be regarded as professional concept conveying, and PB can be regarded as popularized concept conveying. Furthermore, in terms of concept conveying, TSs in xMOOC forums are supposed to convey professional concepts in a popularized way because the audiences of TSs are large in scale and also have prior academic knowledge. In this vein, the linguistic features of SL and PB may form a spectrum of linguistic professionality, where TS may share linguistic features with either SL or PB in some aspects and have different linguistic features from SL and PB in other aspects. Additionally, linguistic features are embedded in language, which may also influence students’ interactions in forums. In this vein, the TS interactions in English and Chinese xMOOC forums are compared. In summary, the following two research questions are proposed as follows.

## 4 Research questions

(1) Are different linguistic features observed in conveying concepts in mTS and fTS interactions? Are different linguistic features observed in these interactions in English and Chinese?
(2) In terms of linguistic professionality, do mTS and fTS show different tendencies toward SL or PB?

## 5 Methods

To answer the two research questions, a quantitative research paradigm named measuring linguistic differences (Borin, 2013) was followed, i.e., distance measures are often evaluated by constructing a tree or network. Several metrics of concept conveying in the mTS, fTS, SL and PB groups are extracted and analyzed. More specifically, the metrics used for analysis include existing linguistic indicators and study-defined indicators informed by graph theory, which are used to model and measure concept conveying. Unlike the common approaches of measuring linguistic differences which construct a syntax tree or a genealogical network, some linguistic features of concept conveying are modeled as a concept graph in this study. Finally, several one-way analyses of variance (ANOVA) are adopted to analyze and justify the differences among these groups in terms of the metrics.

### 5.1 Modeling

**Figure 1:**
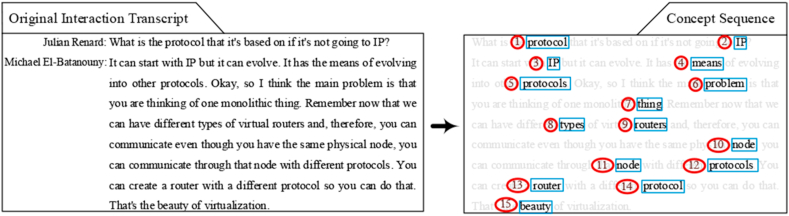
An example of an interaction transcript and its concept sequence.

The concept-conveying procedure was presented by a concept sequence consisting of nouns extracted from interaction transcripts in xMOOC forums. They were arranged in the same order as in the original transcript. Figure 1 shows an example of such a transcript and its extracted concept sequence. The names of the interaction participants can be revealed by searching the original interaction transcript; to protect the privacy of the MOOC students, we used an original interaction transcript published on the Jefferson Science Lecture Series hosted by the Obama Administration instead, which can be fully accessed without registration. The blue boxes frame nominal concepts, and the red circles label the concept positions (i.e., the order of appearance) in a concept sequence.

**Figure 2:**
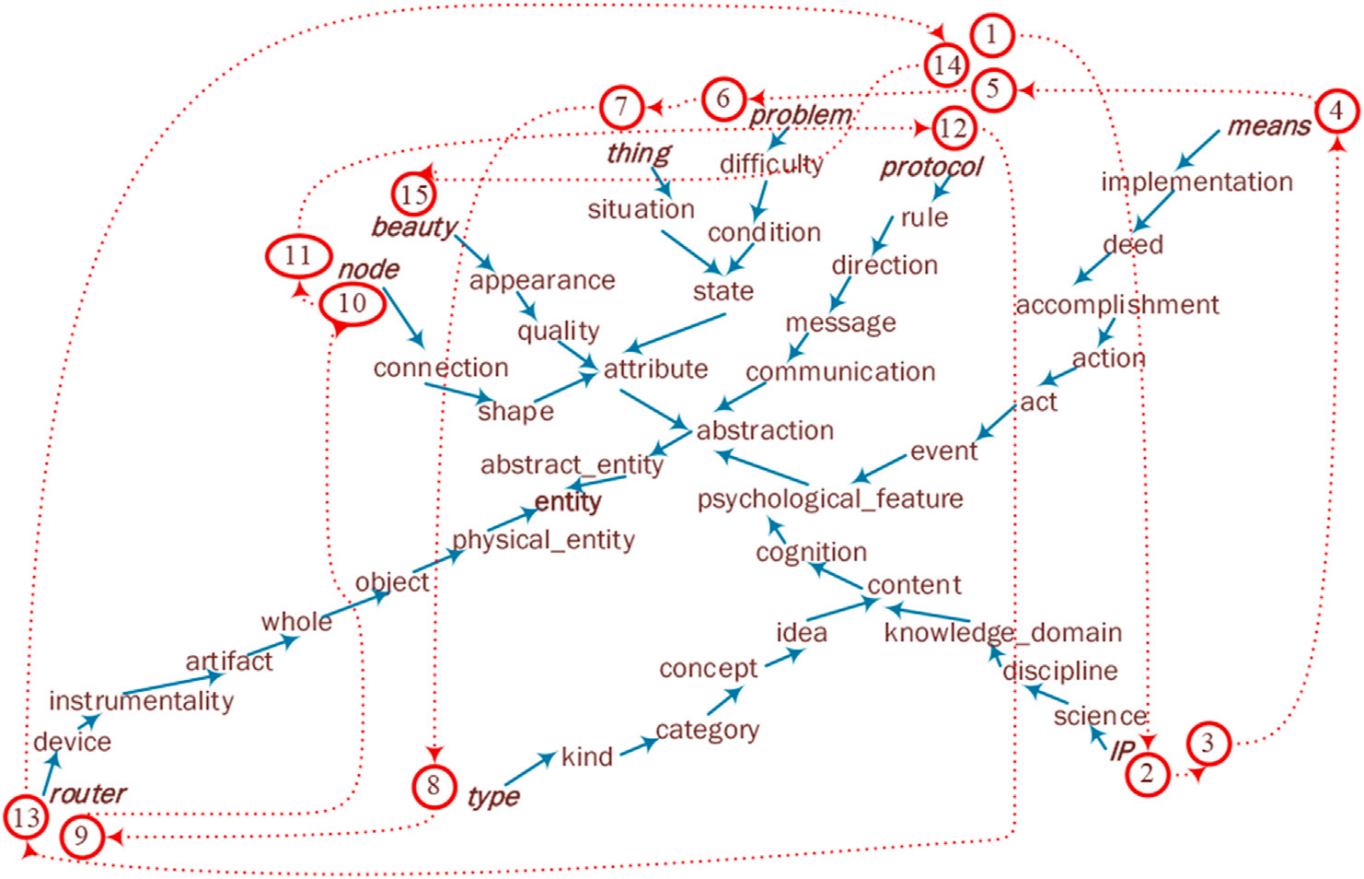
An English example of a concept-conveying graph.

The semantic concrete degree of a noun was represented by its depth in a tree-like structured lexical database, with WordNet (Miller, 1995) used for English and HowNet (Dong and Dong, 2003) used for Chinese. Therefore, concept conveying was represented as a noun chain wrapping around a thesaurus tree, as shown in Figure 2. The graph is called the concept-conveying graph in this study. The leaf nodes are the nouns extracted from the interaction transcript in Figure 1. The nonleaf nodes are the superordinate nouns from the leaf nodes all the way to the root word of WordNet, which is ‘entity’. The red arrows and nearby numbers show the positions of the leaf nodes in the original concept sequence.

#### 5.2 Measurement

The linguistic features of concept conveying were measured at the following two levels: the concept level and the overall level. At the concept level, two study-defined indicators depicted by graph theory were adopted to measure a concept-conveying graph. These indicators were the average (shortest) path length, i.e., APL, summarized by Siew et al. (2019) and the average concrete degree (ACD, also known as the average depth of nodes); more details can be found in Appendix 1.

The depth of a node is the length of the path from the global root entity to that node (Meng et al., 2013). It can be used to measure how concrete a certain concept is in an ‘is-a’ semantic network. In such a semantic network, words are linked to their hypernyms (Bimba et al., 2016). For example, a bird is an animal. Therefore, ‘animal’ is the hypernym of ‘bird’, and ‘bird’ is the hyponym of ‘animal’. The node depth in the ‘is-a’ semantic network also equals the number of hypernym-hyponym levels between this concept and the root of the thesaurus tree. The root is ‘entity’ in WordNet and ‘实体(entity)’ in HowNet. More details can be found in Appendix 1.

A smaller APL indicates that the concepts in a concept-conveying graph are more closely connected on average, which may reduce the intrinsic cognitive load for vicarious learning (Sweller et al., 1998). A larger ACD indicates that the concepts in a concept-conveying graph are more concrete on average, and it may contain fewer instances to allow more students to participate.

At the overall level, the following two common existing linguistic indicators were adopted to measure the entire transcript: readability (Klare, 1974; Chen and Meurers, 2019; Pancer et al., 2019) and semantic overlap degree (Banerjee and Pedersen, 2003; Holi and Hyvönen, 2005).

The readability of transcripts was measured by Flesch–Kincaid Grade Level^2^ (FKGL) for English (McNamara et al., 2014) and Grade Level^3^ for Chinese (Yeh, 2014). A larger FKGL and GL indicate a more difficult transcript for vicarious learning in English and Chinese, respectively. More details about FKGL and GL can be found in Appendix 1.

The semantic overlap degrees of the transcripts were measured by LSASS1 in Coh-Metrix (McNamara et al., 2014). LSASS1 refers to latent semantic analysis on semantic overlap across adjacent sentences (MacArthur et al., 2019); the semantic overlap between adjacent sentences is calculated by a natural language process approach, i.e., latent semantic analysis (Landauer et al., 1998). A larger LSASS1 indicates more semantic overlap in a transcript. A reasonable degree of semantic overlap indicates that a transcript is logically coherent, which lays the foundations for encouraging more responses. Coh-Metrix can now provide LSASS1 values for both English^4^ and Chinese^5^ transcripts. More details can be found in Appendix 1.

These measured outcomes of mTS and fTS are used for comparisons based on the same measures of SL and PB in the same language. To illustrate the comparison clearly, each comparison is presented in a spectrum style figure in the following way: two control groups (SL and PB) are marked in fixed positions; the locations of mTS and fTS markers are determined by their relative positions to SL and PB. The formulas of the relative positions are detailed in the two rightmost columns of Table 5 and Table 6 in Section 6: Results. If Group A were much closer to SL than to PB on a certain indicator, we would say that Group A had a tendency toward SL rather than toward PB on this indicator, or in brief, Group A was more similar to SL than PB.

##### 5.3 Workflow

**Figure 3:**
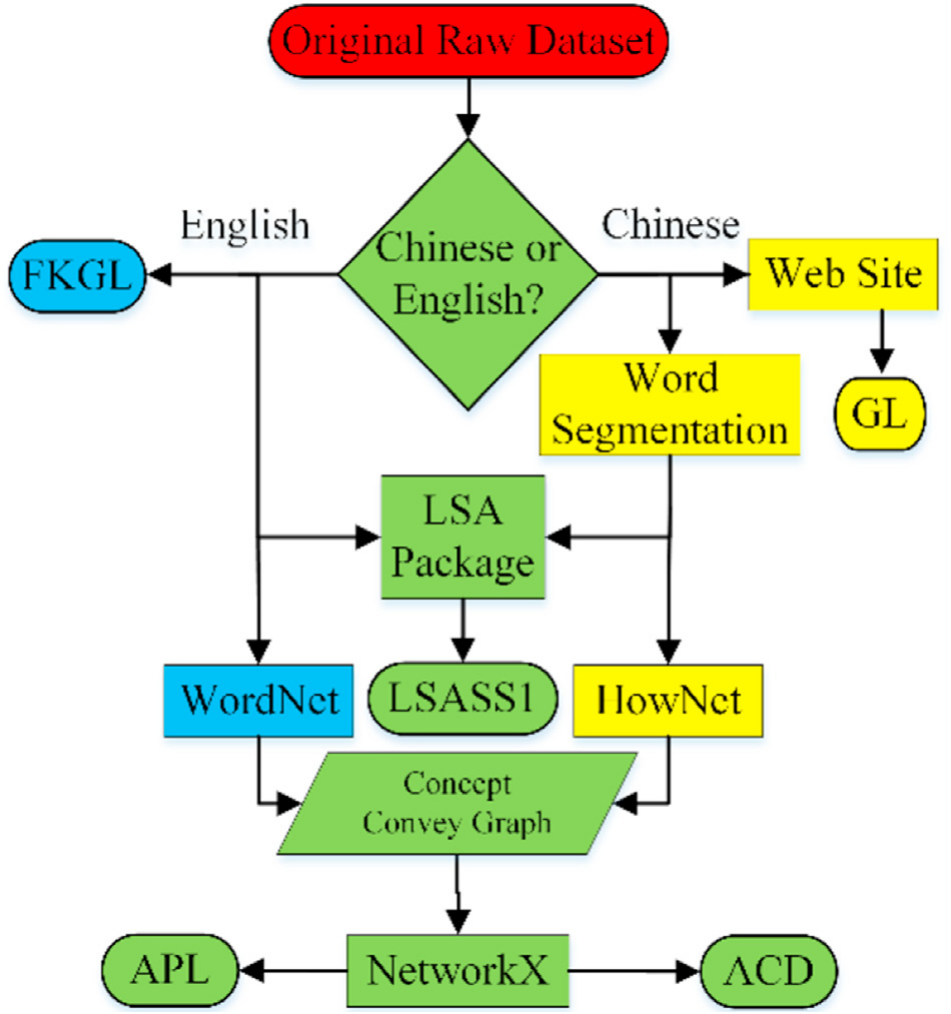
Data processing workflow.

The data processing workflow, including modeling and measurement, is illustrated in Figure 3. This figure summarizes the major processing steps in Subsection 5.1 and 5.2. It is presented according to the method of Goldstine & Neumann, e.g., using a rounded rectangle to indicate a start or an end and using a rhombus to indicate a decision. More technical details are as follows.

The whole workflow was implemented by Python; however, it could also be implemented by C#. Each interaction transcript with screen names removed is treated as a whole, original, and raw data unit to be further processed. Since both English and Chinese have a mature formula or an application to calculate FKGL or GL, these two indicators can be measured very easily. Note that the website in Figure 3 can be found in footnote 10.

However, since English uses spaces to separate words while Chinese does not, the other 3 indicators in Chinese have to be measured after word segmentation is performed (note that there is already an alternative that does not segment Chinese words, and its performance is also good; however, we followed a “traditional” approach in this study). A Chinese natural language process (NLP) package Jieba was imported to our integrated development environment, PyCharm. More specifically, one of its modules, “posseg”, i.e., abbreviated name for part-of-speech & segmentation, was imported to segment the transcripts into words and identify nouns for later processing.

Gensim, another Python library for topic modeling, document indexing and similarity retrieval with large corpora, was imported to calculate LSASS1. More specifically, its modules named “corpus” and “models” were imported to map each sentence of the transcript into an LSA vector. Gensim is also a backup NLP package for the unexpected downtime of the Coh-Metrix official site.

The measurement of the remaining two indicators requires the help of the following NLP tools: HowNet (Chinese) and WordNet (English). Both of these tools have been supported by Python in recent years (note that an old WordNet version also includes a Chinese corpus, but HowNet offers better Chinese support). “OpenHowNet” and “nltk.corpus.wordnet” are their respective package/module names to import when the intermediate output is a concept-conveying graph. NetworkX is the last major package to import to organize the nodes and edges in the concept-conveying graph, based on which APL and ACD are calculated.

##### 5.4 Sampling

Holtz et al. (2012) suggested a practical guide for analyzing Internet forums, with two examples of their own. According to the procedure in their second example, four steps about sampling were extracted and rephrased by us, as follows.

Step 1 identifying the forums based on size, group and content specificity.

Step 2 identifying fields of interest, which further guide the sampling of postings and threads from the forum sections and sub-sections.

Step 3 balancing the proportion of material of each theme (i.e., the interest in Step 2) across boards and sampling subsections for further analysis.

Step 4 setting up criteria to enroll threads as samples, say threads more than 10 posts responding to the initial.

Similar to Wen et al. (2014), Harrak et al. (2019), the details about how a post or a thread was randomly chosen to be a sample was omitted in (Holtz et al., 2012). In our study, it was made clear that a uniform sampling technology was adopted. That was, eligible threads were ordered in a queue by the timestamps of their initial posts and indexed by natural numbers starting from 1. A random number generator whose range was set the same to these indexes was applied, to make sure that each of these threads had an equal chance to be extracted as a sample. In the following subsection 5.5, these four steps were annotated in brackets in the corresponding sentence.

##### 5.5 Datasets

The interaction transcripts were divided into two groups according to their language (English or Chinese). In each group, there were three datasets from different scenarios (TS, PB, and SL). Permission was granted by an Institutional Review Board (IRB) to collect these interaction transcripts from open websites. These transcripts were collected from 2017-2018.

Two ethic issues were addressed before the permission was granted by IRB. The first issue was the protection of personal identity. The second issue was the respect to data ownership. For the personal identity issue, we anonymized the student's screen name in the examples shown in our manuscript. During data preparation, after we made sure that there was not a screen name appearing in more than one sample (balancing the samples), screen names in an interaction transcript were filtered out, and only the content was passed to essential processing steps. For the data ownership issue, MOOC platforms in United States (e.g., EdX) and in China (e.g., icourse163) had different clauses on the ownership of data by their respective terms of service. EdX reminds MOOC students that EdX and EdX members (universities which run courses on EdX, and one of authors of this manuscript is affiliated with such university) may use their posts to support scientific research in the privacy policy of EdX, for example, in the areas of cognitive science and education. Whereas icourse163 warns MOOC students that their information (including posts) appears in public online area where others can also read is not protected in the privacy policy of icourse163. Although the ownership of posts on websites is actually a cutting-edge academic problem in the field of law, in practice it depends on the specific contract made between the content producer and the service provider. In our study, both terms of service of English and Chinese MOOC platforms pledge that our research conduct is consistent with academic ethics.

In the English TS interaction group, the transcripts were collected from a well-known xMOOC community, EdX^6^ (Sampling Step 1). EdX labels two kinds of posts in its course forum: question posts (assumed to be answered by the course team and other students), and discussion posts (assumed to be shared among students). It is easy for viewers to identify a question post or a discussion post by its icon. For a question post, if it is answered by a teaching staff, it will be further annotated, which is helpful to viewers and us. We dived into question posts (Sampling Step 2). Thirty-two online courses were selected from 19 disciplines, such as science, engineering, humanities, art and culture, education, business, and social science. In each course, a TS interaction that started a discussion thread was randomly extracted from its forum (Sampling Step 3 and 4). Please note that Sampling Step 4 applied here did not use a threshold as a criterion, but used a condition: a discussion thread led by a TS interaction, which was a content-related pair involving one question from a student and one immediate answer from a teaching assistant (defined in Section 1: Introduction). Therefore, there were 32 TS interactions involved in our English case. The English SL group, which consisted of 32 Q&A transcripts from 22 disciplines, was randomly extracted from the Jefferson Science Lecture Series hosted by the Obama Administration^7^. In the English PB group, 31 Q&A transcripts of different spokespersons were extracted from routine press briefings hosted by the Trump Administration^8^ that focused on international politics.

For the Chinese groups, similar procedures were followed. There were 32 TS interaction transcripts extracted from a well-known Chinese xMOOC community, icourse163^9^ (Sampling Step 1). Different from labeling question posts and discussion posts in EdX, icourse163 divides course forum into three subsections: course team's reply subsection, course discussion subsection, and general discussion subsection. We dug in course team's reply subsection (Sampling Step 2). In each course, a TS interaction that started a discussion thread was randomly extracted from its forum (Sampling Step 3 and 4). The extraction procedure was the same as the English counterpart. The courses were from 12 disciplines, such as physics, chemistry, geography, medicine, energy, and management. The Chinese SL group consisted of 32 Q&A transcripts from 17 disciplines. The transcripts were extracted from the Science Communication China channel^10^, and some of them were archived in docin^11^ and doc88^12^. In the Chinese PB group, 32 Q&A transcripts of different spokespersons were extracted from routine press briefings hosted by the Chinese Ministry of Foreign Affairs^13^ and focused on international politics.

In both the English and Chinese TS interaction groups, the thread length refers to the number of responses to a TS interaction in a discussion thread. The TS groups were divided into two subgroups by the median thread length (English: 3; Chinese: 1). The subgroup with longer thread lengths was called the mTS interaction group, while the subgroup with shorter thread lengths was called the fTS interaction group. Examples are shown in Appendix 3.

Although randomly extracted from xMOOC forums, the transcripts in the mTS interaction group were checked again manually to ensure that the responses are not superficial in general. The term superficial here means that the responses are not long enough to illustrate diverse and meaningful thoughts. Moore et al. (2019) found that average word counts per post were approximately 50–60, while Joksimović et al. (2015) found that average word counts per post were approximately 20–40 in social media (e.g., Facebook and Twitter). Technically, in this vein, short responses with less than 5 words were labeled ‘superficial’, although manual correction was necessary. If the number of short responses (e.g., ‘agreed’, ‘disagreed’, or ‘thumbs up’) in an mTS interaction were over 50%, this mTS interaction was replaced by a different interaction extracted randomly from the xMOOC forums that had responses more than the median thread length. Additionally, if there were too many responses of an mTS interaction simply because the topic was controversial or offensive, this mTS interaction was handled in the same manner as the interaction with ‘superficial’ short responses.

In addition, if the responses in a TS interaction contain several posts that were copied-and-pasted, which were very rare (because some students wanted to increase their discussion scores by cheating when the course required participation in the forums), the redundant ones were not taken into account, or the entire discussion thread could be even excluded from the samples. The basic statistical information of the qualified samples, together with that of the PB and SL interactions, is listed in Table 1.

**Table 1:** Common metrics of the four groups.

	English			Chinese		
	thread length	sentences /piece	words /piece	thread length	sentences /piece	words /piece
mTS	5.44 (2.34)	13.62 (5.74)	172.81 (78.15)	2.44 (2.03)	4.88 (2.06)	180.44 (69.06)
fTS	1.13 (0.96)	13.81 (6.45)	183.31 (84.99)	0.25 (0.45)	3.94 (1.57)	140.38 (57.25)
PB	-	13.2 (3.6)	101.5 (29.7)	-	14.1 (5.2)	322.9 (107.5)
SL	-	13.1 (6.8)	256.1 (122.2)	-	11.3 (6.7)	497.5 (229.5)

Note. PB and SL do not have thread lengths in either of the two languages. The thread length in an ordinary English xMOOC is 2.68 (SD = 4.49), which is calculated from a general peer-supported forum explored by Onah et al. (2014). Brinton et al. (2014) found that the average thread length in over 73 xMOOCs is 4.98 (SD = 8.65). The thread length in an ordinary Chinese xMOOC has not yet been found. Instead, we use the statistics of (Uijl et al., 2017) as references. They found that the number of responses in the discussion threads of small private online courses (SPOC) was 1.6 (SD = 2.8).

## 6 Results

### 6.1 Comparisons between mTS and fTS

As shown in Table 2, mTS has a significantly smaller ACD and FKGL than fTS in English. These results indicate that in English xMOOC forums, the concepts of mTS interactions are significantly more abstract than those of fTS, and mTS is significantly less difficult to read than fTS. Table 2 also shows that mTS interactions have significantly smaller LSASS1 values than those of fTS in Chinese. This indicates that mTS has significantly less overlapping semantic information than fTS in Chinese xMOOC forums. APL is the only indicator in which significant differences are not observed between mTS and fTS in either language. This indicates that mTS and fTS have no significant difference in concept connectivity.

**Table 2:** Measured indicator values of mTS and fTS with t test results.

	English				Chinese			
	APL	ACD	FKGL	LSASS1	APL	ACD	GL	LSASS1
mTS	5.33 (1.78)	7.00 (0.44)	9.23 (2.11)	0.19 (0.10)	2.79 (0.47)	3.42 (0.38)	4.75 (0.86)	0.26 (0.14)
fTS	5.19 (1.88)	7.28 (0.16)	10.66 (1.81)	0.20 (0.10)	2.86 (0.69)	3.43 (0.20)	4.94 (1.06)	0.41 (0.13)
*t*	0.216	-2.015∗	-2.058∗	-0.283	-0.335	-0.106	-0.557	-3.141∗∗

*Note.* The values in brackets are standard deviations, and the values outside brackets are average values. ∗∗p < 0.01, ∗p < 0.05. Since concept conveying is modeled into a concept sequence, as illustrated in Figures 1 and 2, the mean and standard deviation of ACD are calculated by the positions in the concept sequences. For example, the no. 1 position corresponds to a mean of the concreteness degrees of 16 concepts in the English mTS group. Each concept in these 16 concepts is the 1^st^ concept in the concept consequence extracted from one distinct transcript sample in the English mTS group.

### 6.2 Comparisons between SL and PB

Table 3 shows that SL has a significantly larger APL, FKGL, and LSASS1 than PB, and SL has a significantly smaller ACD than PB in English. These results indicate that the concepts of SL are significantly less connected and significantly more abstract than those of PB in English at the concept level. At the overall level, these results also indicate that SL has significantly more overlapped semantic information than PB and is significantly more difficult to read. In other words, SL conveys more complex concepts than PB in English in a difficult but coherent way. The results also suggest that the four linguistic features can be adopted to differentiate English SL and PB in the spectrum of linguistic professionality.

**Table 3:** Measured indicator values of SL and PB with t test results.

	English				Chinese			
	APL	ACD	FKGL	LSASS1	APL	ACD	GL	LSASS1
SL	6.5 (1.6)	6.95 (0.42)	10.0 (4.2)	0.14 (0.06)	2.99 (0.66)	3.74 (0.28)	4.63 (0.61)	0.34 (0.09)
PB	4.6 (1.2)	7.33 (0.56)	4.8 (1.2)	0.10 (0.06)	2.85 (0.63)	5.12 (0.22)	4.31 (0.93)	0.21 (0.05)
*t*	5.319∗∗∗	-3.053∗∗	6.635∗∗∗	2.645∗	0.868	-21.923∗∗∗	1.628	7.143∗∗∗

*Note.* The values in brackets are standard deviations, and the values outside brackets are average values. ∗∗∗p < 0.001, ∗∗p < 0.01, ∗p < 0.05.

In Chinese, SL and PB show significant linguistic differences in ACD and LSASS1 but also demonstrate insignificant differences in APL and GL. Furthermore, as shown in Table 3, SL has a significantly smaller ACD and significantly larger LSASS1 than PB in Chinese, which is consistent with the results in English. These results indicate that SL in Chinese conveys more abstract concepts in a more coherent way than PB. However, unlike those in English, SL and PB in Chinese have similar levels of concept connectedness and readability. The results suggest that only ACD and LSASS1 can be adopted to differentiate Chinese SL and PB for linguistic professionality.

### 6.3 The tendency of mTS and fTS toward linguistic professionality

The one-way ANOVA results among the four groups are shown in Table 4. For carrying out follow-up comparisons, a Holm-Sidak method (Holm, 1979; Sidak, 1967; Quinn and Keough, 2002) was used to identify significantly differing groups at a significance level of 0.05. The results indicate that both mTS and fTS in English tend to SL in terms of FKGL and LSASS1, suggesting that the concepts in English xMOOC forums are conveyed in a way more like scientists. However, fTS may tend to PB in APL, implying that less connected concepts in English xMOOC forums may result in fewer interactions.

**Table 4:** Multiple comparisons among four groups (mTS, fTS, SL, and PB).

	English				Chinese			
	APL	ACD	FKGL	LSASS1	APL	ACD	GL	LSASS1
*F*	8.018	4.845	24.713	8.427	0.461	238.208	2.279	18.454
*MSE*	2.452	0.200	7.758	0.00571	0.394	0.0728	0.721	0.00952
*p*	<0.001	0.004	<0.001	<0.001	0.710	<0.001	0.085	<0.001
*SL-tendency*	-	-	Both	Both	-	Both	-	fTS
*PB-tendency*	fTS	-	-	-	-	-	-	mTS

*Note*. The last two rows present which groups (fTS/mTS) have a tendency toward the control groups (SL/PB). By following the definition on tendency in the last paragraph of Subsection 5.2: Measurement, some commonly used rules for transforming significant differences into tendency are also adopted, which grantees that such tendency is also at some significance level. For example, if Group A is significantly different from SL but not from PB, Group A will be labeled “PB-tendency”. Another example is that if Groups A and B are both significantly different from SL and PB, their relative positions to SL and PB will be taken into consideration to help make a tendency decision. In addition, “Both” represents fTS and mTS, while “-” indicates that neither group has the tendency.

**Table 5:** Relative positions of the measured indicator values of the four English groups.

	mTS	fTS	SL	PB	(mTS-SL)/|SL-PB|	(fTS-PB)/|SL-PB|
APL	5.33	5.19	6.5	4.6	-0.616	0.311
ACD	7.00	7.28	6.95	7.33	0.132	-0.132
FKGL	9.23	10.66	10.0	4.8	-0.148	1.12
LSASS1	0.19	0.20	0.14	0.10	1.25	2.50

**Table 6:** Relative positions of the measured indicator values of the four Chinese groups.

	mTS	fTS	SL	PB	(mTS-SL)/|SL-PB|	(fTS-PB)/|SL-PB|
APL	2.79	2.86	2.99	2.85	-1.429	0.0714
ACD	3.42	3.44	3.74	5.12	-0.232	-1.217
GL	4.75	4.94	4.63	4.31	0.375	1.969
LSASS1	0.26	0.41	0.34	0.21	-0.615	1.539

The Chinese xMOOC forum results show a different story. As shown in Table 4, both mTS and fTS tend to SL in ACD, suggesting that the concepts conveyed in Chinese xMOOC forums are more abstract in a way similar to scientists. However, in terms of LSASS1, mTS may tend to PB, while fTS may tend to SL. These results imply that when the coherence of concepts conveyed in Chinese xMOOC forums become more like scientists, the interactions may decrease.

### 6.4 Relative positions of mTS and fTS towards SL and PB

As mentioned above, SL and PB can be used as two ends of a ‘ruler’ to highlight mTS/fTS. To illustrate the positions of these four groups, the name tags ‘PB’ and ‘SL’ are fixed on a horizontal numerical axis for a certain indicator, and the name tags ‘mTS’ and ‘fTS’ can slide along the axis according to their positions relative to PB and SL on that indicator. Such relative positions are proportional to the ratio of the distance between mTS/fTS and PB/SL over the distance between SL and PB, as presented in the two rightmost columns of Table 5 and Table 6.

**Figure 4:**
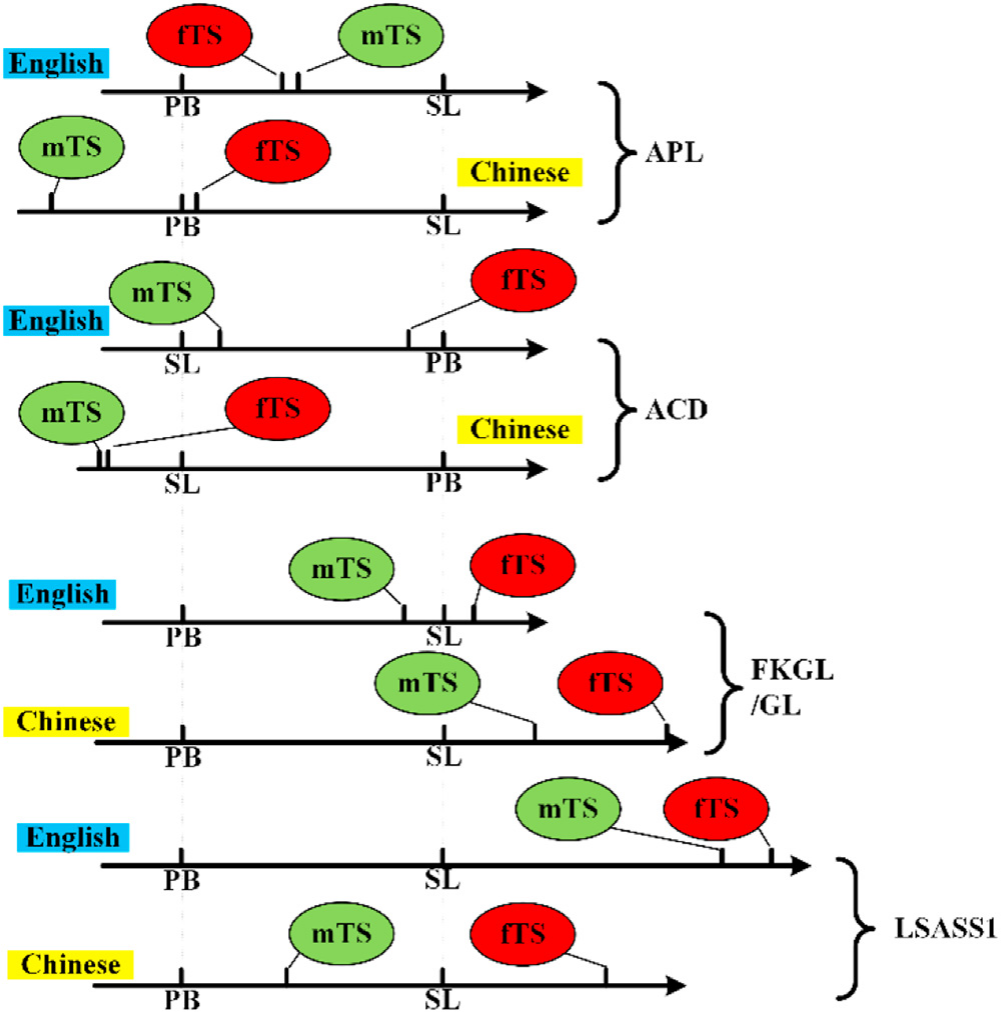
Relative sizes of (mTS, fTS) and (SL, PB).

The relative positions of (mTS, fTS) toward (SL, PB) are shown in Figure 4.

For clarity and comparison convenience, the values of the marks on the axes are omitted, but the distances between the marks are proportional to the average sizes in Table 2. To perform a comparison on the same base over different languages, the values of PB and SL on each axis are scaled to the same range, which works as a fixed ruler. The corresponding mTS or fTS is scaled according to its size relative to the sizes of PB and SL in Table 2.

Notably, among the 8 axes, mTS is on the left side of fTS in all cases except for the English APL axis. The English mTS with a slightly larger APL indicates that the English mTS is a slightly difficult piece material for vicarious learning, which fits with the ‘desirable difficulty’ recommended by Metcalfe (2011) mentioned in Section 2: Literature review. However, as indicated by Table 2, such differences are not significant.

Additionally, mTS tends to be much closer to PB than fTS at the overall level (i.e., FKGL/GL and LSASS1) in both languages. In fact, PB works as a strong magnetic pole attracting mTS closer than fTS on the overall level.

We also find that mTS tends to be farther away from PB than fTS at the concept level (i.e., APL and ACD) in both languages. In fact, PB also works as a strong magnetic pole at this level, but this time, it pushes mTS further away than fTS.

## 7 Discussion

In this section, the discussion on the results is presented, and then the implications of these results are presented, and the discussion on the connections between our study and existing studies are presented at last.

### 7.1 On the results

In this paper, SL and PB are assumed to be two different extremes of linguistic professionality, where the aim of one is to convey concepts in front of a relatively small academic circle and that of the other is to convey concepts in front of a relatively large population. By using common glossaries in graph theory and well-known linguistic features, we found that linguistic features of concept conveying in SL and PB have consistent differences in English and Chinese. These differences imply that these two different extremes may require and evolve two different and relatively fixed styles of linguistic features in terms of concept conveying. TS interactions can be treated as a combination of the two extremes; i.e., they convey academic concepts to a median population (i.e., usually larger than that of SL but smaller than that of PB). With the help of SL and PB, which may form a spectrum of linguistic professionality and serve a function similar to that of pH test strips used in chemical laboratories, mTS is found to be closer than fTS to the PB end at the overall level and closer than fTS to the SL end at the concept level (including mTS on the left of fTS on the Chinese ACD axis).

The only exception is that mTS is farther away from SL than fTS on the Chinese APL axis. Although such an exception is only on the average sense, it indicates that different from English, the concepts conveyed in mTS are more closely connected than those conveyed in fTS in Chinese on average. When concepts in a piece of learning material are closely connected on average, this piece of learning material may be complicated on average. In other words, the students tended to respond to TS interactions with more complicated themes in Chinese forums.

To verify the generality of our findings in TS interactions, a replication of our studies in student-student (SS) interactions is extended in Appendix 2.

### 7.2 On the implications

The results support the following two-step practice for teaching assistants to encourage more responses: they should (1) convey concepts in a more concrete way first and then (2) sustain encouragement by conveying more abstract concepts. Before we relate these two suggestions to the existing literature, we illustrate the two-step practice by mTS and fTS examples found in the transcript samples.

The aim of the first step is to attract more participants at the beginning by having teaching assistants act as motivators, as in ICAP theory (Chi et al., 2018). ICAP, which is short for interactive, constructive, active and passive engagement, is a theory of active learning that differentiates students’ engagement based on their responses. ICAP recommends that supportive lectures plus guided discussions are exemplary circumstances for active engagement, where students are encouraged and tutors (including teaching assistants in xMOOC forums) play the roles of motivators and guides.

The aim of the second step is to provide more opportunities to extend the concepts conveyed by the initial TS interaction. A more abstract concept may mean obscurer learning material, but it may also be more inclusive for different semantic instances than concrete ones. Therefore, more diverse responses, which resonate with this abstract concept, may also be potentially encouraged. This strategy is effective for students with high self-efficacy (Honicke and Broadbent, 2016). In this step, tutors act as facilitators, in accordance with ICAP theory (Chi et al., 2018). Discussions such as seminars facilitated by tutors are exemplary circumstances for constructive engagement. In practice, the above two steps can also be mixed into one scheme in which obscure concepts are wrapped in friendlier coats. Interactions in high or low spirits during PB and SL in native languages can be taken as references for teaching assistants (or new teachers) to either learn from or avoid.

Wong (2021) surveyed a total of 123 language MOOCs from the major MOOC platforms. This survey found that pedagogies adopted by current language MOOCs do not differ substantially from conventional distance language learning. It is reasonable to infer that pedagogies adopted by other non-language MOOCs are more or less like the above phenomenon in language MOOCs as well. Although the large student base of MOOCs is a promising opportunity to develop students' proficiency, instructors reported that they are short of time (Khalil and Ebner, 2015). However, our results implied that teaching assistants or instructors do not have to spend more time on engaging in learning community, but can attract more responses by elaborating their replies to students' questions: using concreteness to clarify students' confusion and using abstraction to inspire (or include) more ideas (or posts). It might be an art for a new hand or a young teaching assistant to balance the professionality and popularization of concepts conveyed, but it is believed that once such art is mastered, it will help to improve the students’ content-related participations in xMOOC forums without extra time cost of the course team.

### 7.3 On the connections

With regard to theoretical connections with existing studies, measuring human thinking by their output (e.g. posts in forums) has a long history. One of recent studies is that Moore et al. (2019) found that the number of words found in LIWC dictionaries are positively associated with cognitive processing (e.g. ‘think’, ‘because’, ‘odd’ or ‘perhaps’) while analytical thinking (formal, logical, and hierarchical thinking patterns) and clout (the relative social status, confidence, or leadership that people display through their writing or talking) are negatively associated. Different from counting words in LIWC or comparing some combination of words in LIWC with the help of natural language processing technologies, our study counts the word (concepts, more specifically) level in a lexical database which is organized like a tree. The concept level, rather than the concept itself, can give us specific information about relative positions of two concepts at the concreteness aspect.

In this study, we also measured other linguistic features in two different layers: concept level, and overall level. Dowell et al. (2017) also investigated two aspects of MOOC students’ language (the depth of learning and the content-relatedness of posts), though these two aspects were not organized in a layered structure. A second similarity between Dowell et al. (2017) and our study is that both studies used FKGL to measure the readability of a transcript.

In order to examine the professionality of TS interactions, our study introduced transcripts in political briefings and scientific lectures as samples for two ends of professionality. It is exciting to find that Candarli (2021) also adopted an axis with two opposite directions to label the relative positions of involved samples, though she did not introduce additional samples as a foil. Candarli (2021) also measured multiple linguistic characteristics of online academic forum posts written by L1 (first language, or native language) English students and L1 Chinese students. Although the linguistic characteristics she measured were different from the metrics of ours, one of phenomena found by her (L1 Chinese writer's posts included more elaborated discourse than L1 English writers' posts) looked related to one of ours (the students tended to respond to TS interactions with more complicated themes in Chinese forums). Although “elaborated” and “complicated” were different to some extent, they shared similar meanings on “sophisticated”. These two associated phenomena may probably be attributed to that Chinese students tend to use long sentences when they participated in an online academic discussion, so those long sentences may have more chances to be elaborated or complicated, even if they turned to use English in a UK university. Interested readers may go for Example 5 and 6 in Candarli (2021), and examples in our appendix 1. Although the explanation behind these two associated findings needs to be further examined and it is beyond the scope of this paper, we believe it is worth being investigated later.

At last, concept conveying embedded in a questioner-respondent interaction reveals a means for measuring the cognitive load of students observing this interaction. Cognitive load theory argues that intrinsic cognitive load is determined by an interaction between the nature of the material being learned and the expertise of the learners, which is assumed to be constant (Sweller et al., 1998). Currently, intrinsic cognitive load is usually measured by scales with subjective questions, behaviors or participant performance (Paas et al., 2003), e.g., reading time and eye tracking. Our concept-conveying graph can be regarded as a different attempt to measure the cognitive load of learning material based on its linguistic features.

## 8 Conclusion

In this paper, the linguistic features of teaching assistant-student xMOOC interactions were examined to investigate how concepts were conveyed in such a learning environment. For this purpose, the interaction transcripts in science lectures (SL) and political briefings (PB) were used as control groups to highlight the linguistic features (i.e., concept connectivity, concept concreteness, readability and semantic overlap) of concept conveying in TS interactions with many responses (mTS) and those with few responses (fTS). The results show that mTS and fTS demonstrate different concept conveying tendencies toward SL and PB in terms of the professionality entailed in linguistic features in two languages, i.e., English and Chinese. The linguistic differences are visualized by placing the indicators of features in a relative manner. By this way, some qualitative data is qualified to some extent. The results suggest that in both languages, teaching assistants may use a two-step practice involving the use of concept-conveying strategies to stimulate more follow-up responses in xMOOC forums, i.e., (1) convey concepts in a more concrete way first and then (2) sustain encouragement by conveying more abstract concepts. Similar findings can also be observed in a larger student-student interaction dataset, as in Appendix 2.

Although the large student base of MOOCs is a promising opportunity to develop students' proficiency, instructors reported that they are short of time (Khalil and Ebner, 2015). However, our results implied that teaching assistants or instructors do not have to spend more time on engaging in learning community, but can attract more responses by elaborating their replies to students' questions: using concreteness to clarify students' confusion and using abstraction to inspire (or include) more ideas (or posts). It might be an art for a new hand or a young teaching assistant to balance the professionality and popularization of concepts conveyed, but it is believed that once such art is mastered, it will help to improve the students’ content-related participations in xMOOC forums without extra time cost of the course team.

One limitation of this study is that though findings observed in TS samples and the ones observed in a larger student-student interaction dataset are similar, each sample comes from a distinct course. Although this approach can balance the diversity of courses, its small size prevents us from exploring more potential significant differences between mTS, fTS, PB or SL on other metrics. For example, more than 100 metrics of Coh-metrix 3.0 listed in (McNamara et al., 2014) had been tried to measure English samples. Only on two of them (LSASS1 and FKGL), mTS, fTS, PB or SL exhibited significant differences. If we can enlarge our samples (of course with more human labor on identifying content-related threads), the possibility of finding more distinguish metrics (including metrics under other frameworks than Coh-metrix) would increase.

Another limitation of this study is that we did not collect interaction transcripts with the same questions but different answers since this study used interaction transcripts as a whole piece of learning material for observing students. However, our future work will consider more factors, such as how a question is proposed or how an answer is organized, and will consider MOOCs with rich discussions, e.g., FutureLearn. In addition, for those phenomena discovered in this study, more investigations should be conducted to examine related hypotheses. A chat robot based on the characteristics found in this study could be developed to manipulate the variables.

## Declarations

### Author contribution statement

Wang Tai: Conceived and designed the experiments; Performed the experiments; Analyzed and interpreted the data; Contributed reagents, materials, analysis tools or data; Wrote the paper.

Hercy N. H. Cheng: Analyzed and interpreted the data.

Zhiqiang Cai: Contributed reagents, materials, analysis tools or data.

### Funding statement

This work was supported by National Natural Science Foundation of China [61877022, 61937001 & 31600918].

### Data availability statement

Data will be made available on request.

### Declaration of interest's statement

The authors declare no competing interests.

### Additional information

No additional information is available for this paper.

## Figures and Tables

**Table 7:** Common metrics of the four groups

	English			Chinese		
	thread length	sentences /piece	words /piece	thread length	sentences /piece	words /piece
mSS	13.41 (16.00)	8.02 (4.82)	127.59 (91.75)	8.23 (6.30)	3.79 (1.68)	44.86 (33.92)
fSS	1.12 (0.99)	8.99 (5.32)	137.93 (97.57)	1.48 (1.07)	3.55 (1.95)	41.05 (47.66)

**Table 8:** Measured indicator values of mSS and fSS with t test results

	English				Chinese			
	APL	ACD	FKGL	LSASS1	APL	ACD	GL	LSASS1
mSS	7.09 (1.02)	7.07 (0.60)	11.15 (2.53)	0.22 (0.14)	4.58 (0.85)	4.52 (0.85)	5.21 (1.40)	0.33 (0.29)
fSS	7.28 (0.95)	7.14 (0.62)	11.41 (4.82)	0.21 (0.15)	4.39 (0.85)	4.22 (0.82)	5.32 (1.23)	0.44 (0.30)
*t*	-1.109	-0.650	-0.358	0.389	1.217	2.039∗	-0.472	-2.122∗

**Table 9:** Multiple comparisons among the four groups (mSS, fSS, SL, and PB)

	English				Chinese			
	APL	ACD	FKGL	LSASS1	APL	ACD	GL	LSASS1
*F*	45.881	2.441	24.248	8.488	57.559	22.688	6.951	6.316
*MSE*	1.276	0.335	14.847	0.0160	0.623	0.492	1.383	0.0602
*P*	<0.001	0.066	<0.001	<0.001	<0.001	<0.001	<0.001	<0.001
*SL-tendency*	Both	-	Both	Both	Both	fSS	Both	fSS
*PB-tendency*	-	-	-	-	-	mSS	-	-

*Note*. The last two rows present, which groups (fSS/mSS), have a tendency toward control groups (SL/PB). By following the definition of tendency in the last paragraph of Subsection 5.2: Measurement, some commonly used rules of transforming significant differences into tendency are also adopted, which grantees that such a tendency is also at some significance level. For example, if Group A is significantly different from SL but not from PB, Group A will be labeled “PB-tendency”. Another example is that if Groups A and B are both significantly different from SL and PB, their positions relative to SL and PB will be taken into consideration to help make a tendency decision. In addition, “Both” represents fSS and mSS, while “-” indicates that neither group has the tendency.

**Figure 5:**
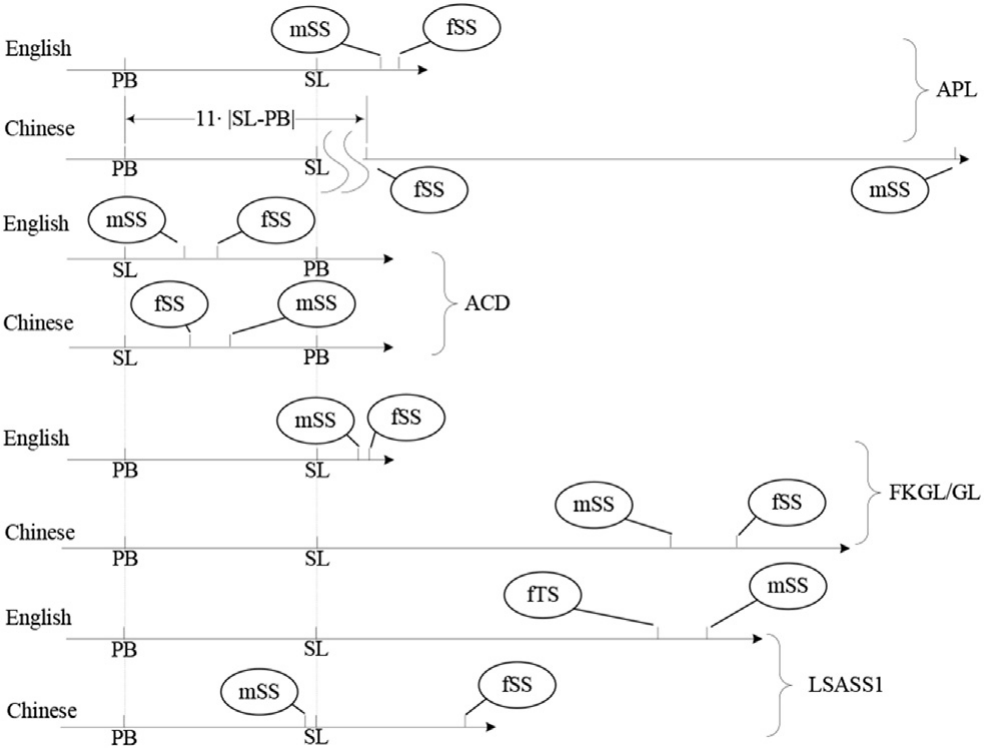
Relative sizes of (mSS, fSS) and (SL, PB).

**Table 10:** Relative positions of the measured indicator values of the four English groups

	mSS	fSS	SL	PB	(mSS-SL)/|SL-PB|	(fSS-PB)/|SL-PB|
APL	7.09	7.28	6.5	4.6	0.311	1.411
ACD	7.07	7.14	6.95	7.33	0.316	-0.5
FKGL	11.15	11.41	10.0	4.8	0.221	1.271
LSASS1	0.22	0.21	0.14	0.10	2	2.75

**Table 11:** Relative positions of the measured indicator values of the four Chinese groups

	mSS	fSS	SL	PB	(mSS-SL)/|SL-PB|	(fSS-PB)/|SL-PB|
APL	4.58	4.39	2.99	2.85	11.357	11
ACD	4.52	4.22	3.74	5.12	0.565	-0.652
GL	5.21	5.32	4.63	4.31	1.813	3.156
LSASS1	0.33	0.44	0.34	0.21	-0.0769	1.769

## Appendix

### Appendix 1 Definitions on APL, ACD, GL, FKGL, and LSASS1

#### Appendix 1.1 APL

Suppose there are *N* nodes (vertices) in a graph, where *d*(*v*_*i*_,*v*_*j*_)is the shortest path length between node *v*_*i*_ and *v*_*j*_. It is the number of steps along the shortest path connecting *v*_*i*_ and *v*_*j*_. APL is the average (shortest) path length between pairs of nodes. It is calculated by $\sum_{i\neq j}d\left(v_{i}{,v}_{j}\right)/N\left(N-1\right)$.

#### Appendix 1.2 ACD

Suppose there are *N* nodes. The depth of a node *n*, depth(*n*), is the length of the path in a thesaurus tree (e.g., WordNet or HowNet) from the global root entity to n. The average concrete degree (ACD, also known as the average depth of nodes) is $\sum_{n=1}^{N}depth\left(n\right)/N$

#### Appendix 1.3 FKGL

FKGL (Flesch–Kincaid grade level) is based on the length of words and length of sentences. FKGL = 11.8S + 0.39 W–15.59. S refers to the mean number of syllables per word, and W refers to the mean number of words per sentence.

#### Appendix 1.4 GL

Grade level (GL) measures the readability of a Chinese transcript. It is calculated by GL = 3.8-2.167×TTRA+0.012×MTLD TTRA is the type-token ratio (All words). It measures the word diversity of a transcript. It is calculated as the ratio of the number of types over the number of tokens. A token is a term in a transcript. Regardless of whether it occurs more than once, it is counted as an independent token. A type is a distinct term in a transcript. If a term occurs more than once, it is only counted as one type. MTLD is the measure of textual lexical diversity. TTRA may decrease with the text length, while MTLD will not. MTLD is calculated as the mean length of sequential word strings in a text that maintain a given type-token ratio value (here .720). The calculation of the MTLD is more complicated than a simple formula. More details can be found in (McCarthy and Jarvis, 2010).

#### Appendix 1.5 LSASS1

LSASS1 is the average LSA similarity between two adjacent sentences. Suppose there are *S* sentences in a transcript. The LSA's cosine-like similarity between the *i*-th sentence and the (*i*+1)-th sentence is LSA(*i*, *i*+1). Then, LSASS1 = $\sum_{i=1}^{S-1}LSA\left(i,i+1\right)/\left(S-1\right)$

### Appendix 2 An extended replication of our studies in student-student interactions

It is rather difficult to collect a large number of qualified TS interactions from xMOOC forums. This is because (1) the TS interactions studied in our work, whose question is asked by a student and the first reply comes from the teaching team, are not common in xMOOCs; and (2) only a limited number of such interactions in each course can be randomly collected so that the bias among courses can be suppressed and courses can cover as many disciplines as possible. For these reasons, we must use to the student-student (SS) interactions that are fairly common in xMOOCs to verify the generality of our findings in TS interactions. Among these SS samples, we performed an extended replication of our studies. Note that in contrast to TS interactions, SS interactions in this extended replication do not involve any teaching team members.

#### Appendix 2.1 Datasets

In the English SS interaction group, one hundred forty transcripts were collected from the same English xMOOC community from which the above TS interaction data were collected, a.k.a., edX. They involved 31 courses from 16 disciplines, including biology, chemistry, climate, computer science, design, economics, finance, literature, management, materials, mathematics, media and medicine.

For the Chinese groups, similar procedures were followed. One hundred thirty SS interaction transcripts were extracted from the same Chinese xMOOC community from which the above TS interaction data were collected, a.k.a., icourse163. They involved 22 courses from 9 disciplines, including botany, chemistry, geography, health, management, mechanics, medicine, physics, and sports.

Similarly, the SS groups were divided into two subgroups by the median thread length (English: 4; Chinese: 4). The subgroup with longer thread lengths was called the mSS interaction group, while the subgroup with shorter thread lengths was called the fSS interaction group. Their basic statistical information is listed in Table 7.

#### Appendix 2.2 Comparisons between mSS and fSS

As shown in Table 8, there are no significant differences between mSS and fSS in the English forum interactions for any of the four indicators. In the Chinese forum interactions, however, the mSS has a significantly larger ACD and a significantly smaller LSASS1 than fSS. These results indicate that in Chinese xMOOC forums, the concepts of mSS are significantly more concrete than those of fSS. They also indicate that mSS has significantly less overlapping semantic information than fSS in Chinese xMOOC forums, which is consistent with the difference we found in the mTS-fTS comparison for the LSASS1 indicator in Table 2.

#### Appendix 2.3 The tendency of mSS and fSS toward linguistic professionality

The results in Table 9 indicate that both mSS and fSS in English tend to SL in terms of APL, FKGL and LSASS1, suggesting that the concepts in the initial SS interaction in English xMOOC forums are conveyed in a way more similar to scientists.

The Chinese xMOOC forum results are different. As shown in Table 9, both mSS and fSS tend to SL in APL and GL, suggesting that the concepts conveyed in the initial SS interaction in Chinese xMOOC forums are similar to scientists in terms of the connectivity and readability. However, in terms of ACD, mSS may tend toward PB, while fSS may tend toward SL. These results imply that when the concreteness of concepts conveyed in the initial SS interaction in Chinese xMOOC forums become more similar to that of scientists, the follow-up replies may decrease. In addition, fSS may tend to SL in terms of LSASS1, whose tendency is the same as fTS in Table 4.

#### Appendix 2.4 Relative positions of mSS and fSS towards SL and PB

Similar to Subsection 6.4: Relative positions of mTS and fTS towards SL and PB, the relative positions of mSS and fSS toward SL and PB are plotted in Figure 5 by the data calculated in Tables 10 and 11.

Unlike the almost complete consistency of the relative positions of mTS and fTS on each indicator for the different languages (Figure 4), the relative positions of mSS and fSS on each indicator are almost exactly opposite for the different languages. For example, when mSS is on the left side of fSS on the APL axis in English, it is on the right side of fSS on the same axis in Chinese. The only indicator where mSS and fSS exhibit the same relative positions in both languages is FKGL/GL (readability). On this indicator, mSS is on the left side of fSS in both English and Chinese.

The “magnetic pole” metaphor used in TS cases becomes complicated in SS cases. In TS cases, there is only one magnetic pole (PB), although either “attracting” or “repelling” depends on the overall level or concept level, regardless of the language. However, in the SS cases, there is still one magnetic pole that now turns into SL, but the “attracting” or “repelling” depend not only on the levels but also the languages. Specifically, in Chinese, SL works as a strong magnetic pole pushing mSS further away than fSS at the concept level (i.e., APL and ACD), and it works as a strong magnetic pole attracting mSS much closer than fSS at the overall level (i.e., GL and LSASS1). The English situation is less complicated: SL works as a strong magnetic pole attracting mSS much closer than fSS on almost all four indicators except LSASS1.

#### Appendix 2.5 Summation

Comparing Tables 4 and 9 shows that both TS and SS interactions have the same SL tendency on FKGL and LSASS1 in English and both fTS and fSS interactions have the same SL tendency on LSASS1 and ACD in Chinese individuals.

The reasons behind each of the above similarities or differences deserve deep exploration in the future. Although such an exploration is beyond the scope of this paper, it is worth noting two promising factors that the hidden reasons may relate to, i.e., language and the teacher's role in learning.

The latter factor has relatively more existing empirical cues than the former. Xu et al. (2020) shows that the behavioral and cognitive engagement of the experimental group with teacher facilitation was significantly higher than that of the control group without teacher facilitation. Additionally, the emerging student leaders show similar positive effects of teacher facilitation toward improving behavioral and cognitive engagement. Nandi et al. (2012) indicates that periodic feedback from instructors is always highly valued by students and keeps the students on track; hence, this feedback is essential. Their results also validate that handing students the responsibility to direct discussion is not always the best option and instructors should be in control of the discussion at all times through an active presence to guarantee the discussion quality. A large-scale and long-term investigation (Webster et al., 2011) finds that only well-trained (in terms of preparedness, deployment, and practice) teaching assistants (TA) can have a positive impact on pupils' (younger than college students') academic progress, as ill-trained TAs do not know how to make the best use of the extended, more frequent interactions with pupils compared with teachers; i.e., ill-trained TAs' interactions fail to foster active pupil participation, which has longer-term implications for creating passive learners. Webster et al. (2011) also finds that a key difference between teacher-to-pupil interactions and (ill-trained) TA-to-pupil interactions is that teachers generally ‘open up’ pupil interactions, whereas the TAs ‘close down’ the interactions, both linguistically and cognitively. The “emerging student leaders” in (Xu et al., 2020) and the ill-trained TA's negative impact in (Webster et al., 2011) may help to explain why SS interaction tendencies are more complicated than TS interaction tendencies.

These studies shed light on exploring the reasons behind the above similarities or differences between TS and SS. However, how this empirical evidence connects to our measurement framework of 4 indicators is still worth exploring in the future.

### Appendix 3 Examples of TS interactions

#### A. Chinese mTS and fTS Examples

A Chinese mTS interaction that initiates 8 responses is presented below. Student: 植物是不能运动，那痒痒树这种植物的这种反应能不能称之为运动呢？(Plants are not supposed to move. Can the response of itch trees be called movement?) Teaching Assistant: 紫薇又称“痒痒树”，紫薇树的木质比较坚硬，最大的特点是枝干的根部和梢部差不多粗细，那样的话就有别于一般树干下粗上细的特点，也就是说，紫薇树的上部比一般的树要重些，也就决定它容易摇晃，当我们用手指挠它的枝干时，摩擦引起的震动很容易通过坚硬的木质传导到枝干的更多部位，于是就容易引起摆动。(The crape myrtle tree is also called “itchy tree”. Its wood is relatively hard. The most distinct feature is that the roots of the branches are as thick as the tips of the branches. This is different from an ordinary tree whose roots are usually much thicker than the tips. Therefore, the tips of the crape myrtle tree are usually heavier than those of other normal trees. This feature causes it easily to shake. When we scratch the branch with our fingers, the vibration caused by friction is easily transmitted to more parts of the branch through the hard wood, so it swings easily.)

This TS interaction begins with a very concrete concept, i.e., itch trees/crape myrtle trees. While the teaching assistant explains why itch trees “fear” itch, more abstract concepts, such as “roots”, “tips”, “wood”, “vibration”, and “friction”, appear in the concept conveying sequence. These concepts are closely connected (roots, tips, and wood are in the concept domain of trees, and vibration and friction are in the concept domain of movement). These abstract concepts can encourage lurkers to associate broadly with their own knowledge or experience because many different types of instances entailed by the abstract concepts can offer many opportunities for association to occur. The close connectivity of these concepts can cause this association to easily occur. Therefore, the description and explanation of the “itch-fear” tree in TS interactions can efficiently remind lurkers of other similar plants, e.g., Mimosa pudica/shy plants. In fact, there is a “shy plant” mentioned in a response to this TS interaction.

A Chinese fTS interaction that initiates a zero response is presented below. **Student**: 天然添加剂为什么是非天然部分？食品添加剂中天然和合成的区别？(Why are natural additives unnatural? What is the difference between natural and synthetic food additives?) **Teaching Assistant**: 大部分天然添加剂分为两类：一是天然提取物，二是以发酵方法制取物。所以说天然添加剂也有非天然部分。(Most natural additives are divided into two categories: one is natural extracts, and the other is made by fermentation. Thus, natural additives also have unnatural parts.)

In terms of the whole piece, this above TS interaction stays at the same abstract level where the student begins. No other concrete concept is introduced during the teaching assistant's explanation. Therefore, there is a lack of instances to illustrate what is a natural additive and how it is made, although the teaching assistant does answer the question literally. Moreover, it fails to utilize examples, which is a commonly used scaffolding to encourage lurkers to respond, unless some lurkers are willing to ask what fermentation is.

#### B. English mTS and fTS Examples

An English mTS interaction that initiates 6 responses is presented below. Student: It is largely our business, forming the whole economy, that are responsible for so many challenges we face. But we cannot solely blame them because, in a sense they are satisfying needs people raise. So I think, profound change has to start at the root of the cycle - the demand of the consumers. How do you change the perceptions and opinions held? Teaching Assistant: Hi, xxx (the screen name of the above student), welcome to the course! I hope it’s a really enriching experience for you! Thank you as well for getting us started in the forum! I think what I’m hearing from you is that a change in values is the most important thing required for sustainable development, on the part of both businesses and citizens (as consumers). Does that sound right? Have you seen any notable examples you’d like to point people towards? I think you’ve got a great point about the need to reform the economy and how it operates, what do you think about the role of other actors, like governments or multilateral institutions like the UN?

Compared with the former Chinese mTS example, this English mTS example apparently demonstrates more than one efficient encouraging strategy. The first and most obvious one is that the teaching assistant begins with setting up a warm atmosphere, such as “Hi, …welcome…, ……Thank you…”. This is not only a warm welcome to this student but also to all lurkers. Of course, using a only warm greeting is not enough. The second strategy that the teaching assistant continues to apply is to “narrow” the discussion down to a more concrete focus. To achieve this, this teaching assistant asks the student to give some examples (“......notable examples you'd like to point”). Then, the teaching assistant hints at other different but related concrete concepts (“role…actor…governments or multilateral institutions…”). These two strategies are quite effective. The following 6 responses not only come from 6 distinct students but also involve many words. More specifically, there are 309, 128, 74, 48, 35, and 12 responding words (from the most to the fewest), respectively. The average number of responding words is 101, which is 58.4% of the total number of TS interaction words: 173.

An English fTS interaction that initiates a zero response is presented below.

Student: It is said in the lesson (around 19:30) that corporations are able to borrow at a lower interest rate than the governments of the countries, because they are more likely to pay it back. Considering that corporations can have bad results and end declaring bankruptcy, on the other hand, governments, in a last resort, can print money to pay their debts. So, why it is more likely to a corporation pay its debts?

Teaching Assistant: Hi, xxx (the screen name of the above student). Please find xxx (the lecturer)'s response below: “Let me clarify my statement. First the borrowing I was referring to was in a reserve currency that neither the corporation nor the government can print (such as an Argentinian corporation or the government of Argentina borrowing in euros). Second, while there are several corporations in the world that can borrow reserve currencies at a lower interest rate than their governments, this is not true generally (only when the government has a very large foreign currency debt and trade deficit, or a history of defaulting). You are quite right that bonds from a sovereign government borrowing in its own currency have no credit risk as the government can print an unlimited amount of its own currency.”

This above fTS example shares the same knowledge domain as the former English mTS example, i.e., economics. Compared with the Chinese fTS example, the content of this English fTS example is much more specific, and it includes more concrete concepts. However, this English fTS example is about a detailed clarification forwarded from the lecturer who is not present in the forum, which makes it so specific that no more related concepts need to be extended. In other words, the purpose of this interaction (clarification) prevents further exploration to develop concepts that are either more concrete or more abstract to allow students to “hop” in. This example demonstrates again that if no more abstract concepts are added in the interaction, there will be fewer responses.

## Footnotes

1 Although the term “linguistic features” appears both in this subsection and subsection 2.3, it is applied in different contexts: the term used in subsection 2.4 is applied to distinguish content-related posts and non-content-related posts; the term used in subsection 2.3 is applied actually within the scope of content-related posts. Due to such differences, we did not merge these two subsections into one.

2 http://cohmetrix.com/.

3 http://210.240.188.161/Chinese_CohMetrix/index.html.

4 http://141.225.41.245/download/cohmetrix2018.zip.

5 http://141.225.41.245/download/cohmetrixcn_release_2018_09_16.zip.

6 https://www.edx.org/.

7 https://2009-2017.state.gov/e/stas/series/index.htm.

8 https://www.whitehouse.gov/briefings-statements/.

9 https://www.icourse163.org/.

10 http://jysh.people.cn/.

11 http://www.docin.com.

12 http://www.doc88.com/.

13 https://www.fmprc.gov.cn/.

## References

[bib1] Almatrafi O., Johri A. (2019). Systematic review of discussion forums in massive open online courses (moocs). IEEE. Trans. Learn. Tech..

[bib2] Anmarkrud Ø., Bråten I., Strømsø H.,I. (2014). Multiple-documents literacy: strategic processing, source awareness, and argumentation when reading multiple conflicting documents. Learn. Indiv Differ.

[bib3] Banerjee S., Pedersen T. (2003). Extended gloss overlaps as a measure of semantic relatedness. Proc. IJCAI.

[bib4] Bimba A.,T., Idris N., Al-Hunaiyyan A., Mahmud R.,B., Abdelaziz A., Khan S., Chang V. (2016). Towards knowledge modelling and manipulation technologies: a survey. Int. J. Inf. Manag..

[bib5] Borin L., Borin L., Saxena A. (2013). Approaches to Measuring Linguistic Differences.

[bib6] Bråten I., Brante E.,W., Strømsø H.,I. (2018). What really matters: the role of behavioural engagement in multiple document literacy tasks. J. Res. Read..

[bib7] Brinton C.,G., Chiang M., Jain S., Lam H., Liu Z., Wong F.,M.,F. (2014). Learning about social learning in moocs: from statistical analysis to generative model. IEEE Trans. Learn. Technol..

[bib8] Brownell S.,E., Price J.,V., Steinman L. (2013). Science communication to the general public: why we need to teach undergraduate and graduate students this skill as part of their formal scientific training. J. Ugrd. Neurosci. Educ..

[bib9] Candarli D. (2021). Linguistic characteristics of online academic forum posts across subregisters, L1 backgrounds, and grades. Lingua.

[bib10] Carroll J.,B. (1964). Words, meanings and concepts. Harv. Educ. Rev..

[bib11] Chen X., Meurers D. (2019). Linking text readability and learner proficiency using linguistic complexity feature vector distance. J. Comput. Assist. Lang. L..

[bib12] Chi M.,T., Adams J., Bogusch E.,B., Bruchok C., Kang S., Lancaster M. (2018). Translating the ICAP theory of cognitive engagement into practice. Cognit. Sci..

[bib13] Chi M.T., Kang S., Yaghmourian D.,L. (2017). Why students learn more from dialogue-than monologue-videos: analyses of peer interactions. J. Learn. Sci..

[bib14] Chiu T.,K.,F., Hew T.,K.,F. (2018). Factors influencing peer learning and performance in MOOC asynchronous online discussion forum. Australas. J. Educ. Technol..

[bib15] Cho M.,H., Cho Y.,J. (2016). Online instructors’ use of scaffolding strategies to promote interactions: a scale development study. Int. Rev. Res. Open Dist. Learn..

[bib16] Cobb T., Boulton A., Biber D., Reppen R. (2015). Cambridge Handbook of Corpus Linguistics.

[bib17] Cohen A., Shimony U., Nachmias R., Soffer T. (2019). Active learners’ characterization in mooc forums and their generated knowledge. Br. J. Educ. Technol..

[bib18] Cox R., Mckendree J., Tobin R., Lee J., Mayes T. (1999). Vicarious learning from dialogue and discourse: a controlled comparison. Instr. Sci..

[bib19] Craig S.,D., Gholson B., Ventura M., Graesser A.,C. (2000). Overhearing dialogues and monologues in virtual tutoring sessions: effects on questioning and vicarious learning. Int. J. Artif. Intell. Educ..

[bib20] Craig S.D., Sullns J., Witherspoon A., Gholson B. (2006). The deep-level-reasoning-question effect: the role of dialogue and deep-level-reasoning questions during vicarious learning. Cognit. InStruct..

[bib21] Cross K., Cross K. (1999).

[bib22] de Bruin W.,B., Bostrom A. (2013). Assessing what to address in science communication. Proc. Natl. Acad. Sci. USA.

[bib23] Dong Z., Dong Q. (2003). Proc.

[bib24] Dorogovtsev S.,N., Mendes J.,F.,F. (2001). Language as an evolving word web. Proc. Roy. Soc. B.

[bib25] Dowell N., M M., Brooks C., Kovanović V., Joksimović S., Gašević D. (2017).

[bib26] Fesel S.,S., Segers E., Verhoeven L. (2018). Individual variation in children’s reading comprehension across digital text types. J. Res. Read..

[bib27] Gillani N., Eynon R. (2014). Communication patterns in massively open online courses. Internet High. Educ..

[bib28] Goshtasbpour F., Swinnerton B., Morris N.,P. (2019). Look who’s talking: exploring instructors’ contributions to massive open online courses. Br. J. Educ. Technol..

[bib29] Graesser A.C., Person N.,K. (1994). Question asking during tutoring. Am. Educ. Res. J..

[bib30] Grant L., Ginther A. (2000). Using computer-tagged linguistic features to describe l2 writing differences. J. Sec Lang. Writ..

[bib31] Gregori E.,B., Zhang J., Galván-Fernández C., Fernández-Navarro F.,A. (2018). Learner support in moocs: identifying variables linked to completion. Comput. Educ..

[bib32] Harary F., Norman R.,Z. (1953).

[bib33] Harrak F., Bouchet F., Luengo V., Bachelet R. (2019). Proc. EDM, Montréal, Canada.

[bib34] Hay D. (2007). Using concept maps to measure deep, surface and non-learning outcomes. Stud. High Educ..

[bib35] Hew K.,F. (2016). Promoting engagement in online courses: what strategies can we learn from three highly rated moocs. Br. J. Educ. Technol..

[bib36] Hew K.,F. (2018). Unpacking the strategies of ten highly rated moocs: implications for engaging students in large online courses. Teach. Coll. Rec..

[bib37] Hew K.,F., Cheung W.,S. (2014). Students’ and instructors’ use of massive open online courses (moocs): motivations and challenges. Educ. Res. Rev..

[bib38] Holi M., Hyvönen E. (2005). Proc.

[bib39] Holm S. (1979). A simple sequentially rejective multiple test procedure. Scand. J. Stat..

[bib40] Holtz P., Kronberger N., Wagner W. (2012). Analyzing internet forums: a practical guide. J. Media Psychol..

[bib41] Honicke T., Broadbent J. (2016). The influence of academic self-efficacy on academic performance: a systematic review. Educ. Res. Rev..

[bib42] Joksimović S., Kovanović V., Jovanović J., Zouaq A., Gašević D., Hatala M. (2015). Proc. ACM LAK, Poughkeepsie, New York, USA.

[bib43] Jonassen D.,H., Beissner K., Yacci M. (1993).

[bib44] Kellogg S.,B., Booth S., Oliver K.,M. (2014). A social network perspective on peer support learning in moocs for educators. Int. Rev. Res. Open Dist. Learn..

[bib45] Khalil H., Ebner M. (2015). How satisfied are you with your mooc? - a research study about interaction in huge online courses. J. Mass Commun..

[bib46] Klare G.,R. (1974). Assessing readability. Read. Res. Quart..

[bib47] Kucer S.,B. (2014).

[bib48] Landauer T.,K., Foltz P.,W., Laham D. (1998). Introduction to latent semantic analysis. Discourse Process.

[bib49] Loizzo J., Ertmer A.,P. (2016). MOOCocracy: the learning culture of massive open online courses. Educ. Technol. Res. Dev..

[bib50] MacArthur C.,A., Jennings A., Philippakos Z.,A. (2019). Which linguistic features predict quality of argumentative writing for college basic writers, and how do those features change with instruction?. Read. Writ..

[bib51] Marshall J., Vashe M. (2008). Mining and making: developing and conveying concepts in art. Art Educ..

[bib52] McCarthy P.M., Jarvis S. (2010). MTLD, VOCD-D, and HD-D: a validation study of sophisticated approaches to lexical diversity assessment. Behav. Res. Methods.

[bib53] McNamara D.,S., Crossley S.,A., McCarthy P.,M. (2010). Linguistic features of writing quality. Writ. Commun..

[bib54] McNamara D.,S., Graesser A., McCarthy P., Cai Z. (2014).

[bib55] Meng L., Huang R., Gu J. (2013). A review of semantic similarity measures in WordNet. Int. J. Hybrid Inf. Technol..

[bib56] Metcalfe J., Benjamin A.S. (2011). Successful Remembering and Successful Forgetting: A Festschrift in Honor of Robert A. Bjork..

[bib57] Miller G. (1995). WordNet: a lexical database for English. Commun. ACM.

[bib58] Moore R.,L., Oliver K.,M., Wang C. (2019). Setting the pace: examining cognitive processing in mooc discussion forums with automatic text analysis. Interact. Learn. Environ..

[bib59] Nandi D., Hamilton M., Harland J. (2012). Evaluating the quality of interaction in asynchronous discussion forums in fully online courses. Dist. Educ..

[bib60] Nastase V., Mihalcea R., Radev D. (2015). A survey of graphs in natural language processing. Nat. Lang. Eng..

[bib61] Onah D.,F.,O., Sinclair J.,E., Bovatt R. (2014).

[bib62] Paas F., Tuovinen J., Tabbers H., van Gerven P. (2003). Cognitive load measurement as a means to advance cognitive load theory. Educ. Psychol..

[bib63] Pancer E., Chandler V., Poole M., Noseworthy T.,J. (2019). How readability shapes social media engagement. J. Consum. Psychol..

[bib64] Poquet O., Dawson S., Dowell N., Proc L.A.K., Vancouver (2017). How Effective Is Your Facilitation? Group-Level Analytics of Mooc Forums.

[bib65] Quinn G., Keough M. (2002).

[bib66] Rakedzon T., Segev E., Chapnik N., Yosef R., Baram-Tsabari A. (2017). Automatic jargon identifier for scientists engaging with the public and science communication educators. PLoS One.

[bib67] Ramesh A., Goldwasser D., Huang B., Daumé H., Getoor L. (2013). Proc. NIPS Data Driven Education.

[bib68] Richter T., Maier J. (2017). Comprehension of multiple documents with conflicting information: a two-step model of validation. J. Educ. Psychol..

[bib69] Rovai A.P. (2007). Facilitating online discussions effectively. Internet High. Educ..

[bib70] Schleppegrell M.,J. (2001). Linguistic features of the language of schooling. Ling. Educ..

[bib71] Shaffer D.,W., Collier W., Ruis A.,R. (2016). A tutorial on epistemic network analysis: analyzing the structure of connections in cognitive, social and interaction data. J. Learn. Anal..

[bib72] Sidak Z.,K. (1967). Rectangular confidence regions for the means of multivariate normal distributions. J. Am. Stat. Assoc..

[bib73] Siew C.,S.,Q., Wulff D.,U., Beckage N.,M., Kenett Y.,N. (2019).

[bib74] Silva A.,D., Dennick R. (2010). Corpus analysis of problem-based learning transcripts: an exploratory study. Med. Educ..

[bib75] Stewart J., van Kirk J., Rowell R. (1979). Concept maps: a tool for use in biology teaching. Am. Biol. Teach..

[bib76] Stubbs M. (1996).

[bib77] Sweller J., van Merrienboer J., Paas F. (1998). Cognitive architecture and instructional design. Educ. Psychol. Rev..

[bib78] Tawfik A.,A., Reeves T.,D., Stich A.,E., Gill A., Hong C., Mcdade J., Pillutla V.,S., Zhou X., Giabbanelli P.J. (2017). The nature and level of learner - learner interaction in a chemistry massive open online course (mooc). J. Comput. High Educ..

[bib79] Tomkin J.,H., Charlevoix D. (2014).

[bib80] Uijl S., Filius R., Ten Cate O. (2017). Student interaction in small private online courses. Med. Sci. Educ..

[bib81] Vellukunnel M., Buffum P., Boyer K.,E., Forbes J., Heckman S., Mayer-Patel K. (2017). Proc.

[bib82] Verhoeven L., Graesser A.,C. (2008). Cognitive and linguistic factors in interactive knowledge construction. Discourse Process.

[bib83] Waks L.,J., Waks L.J. (2016). The Evolution and Evaluation of Massive Open Online Courses: MOOCs in Motion.

[bib84] Wang X., Wen M., Rosé C.,P. (2016).

[bib85] Webster R., Blatchford P., Bassett P., Brown P., Martin C., Russell A. (2011). The wider pedagogical role of teaching assistants. Sch. Leader. Manag.: Former. Sch. Organ..

[bib86] Weinerth K., Koenig V., Brunner M., Martin R. (2014). Concept maps: a useful and useable tool for computer-based knowledge assessment? A literature review with a focus on usability. Comput. Educ..

[bib87] Wen M., Yang D., Rosé C.,P. (2014). Proc.

[bib88] Whitelock-Wainwright A., Laan N., Wen D., Gašević D. (2020). Exploring student information problem solving behavior using fine-grained concept map and search tool data. Comput. Educ..

[bib89] Wilks C., Meara P. (2002). Untangling word webs: graph theory and the notion of density in second language word association networks. Sec. Lang. Res..

[bib90] Wise A.,F., Cui Y. (2018). Learning communities in the crowd: characteristics of content related interactions and social relationships in MOOC discussion forums. Comput. Educ..

[bib91] Wise A.,F., Cui Y., Jin W., Vytasek J. (2017). Mining for gold: identifying content-related mooc discussion threads across domains through linguistic modeling. Internet High. Educ..

[bib92] Wise A.,F., Cui Y., Vytasek J. (2016).

[bib93] Wong B.,T. (2021). A survey on the pedagogical features of language massive open online courses. Asian Assoc. Open. Univ. J..

[bib94] Wong J.,S., Pursel B., Divinsky A., Jansen B.,J. (2015).

[bib95] Xu B., Chen N.-S., Chen G. (2020). Effects of teacher role on student engagement in WeChat-Based online discussion learning. Comput. Educ..

[bib96] Yeh C.,J. (2014).

[bib97] Zhao Q., Varma S., Konstan J.A. (2017).

[bib98] Zhou L., Jiang C., Wu Y., Yang Y. (2015). Conveying the concept of movement in music: an event-related brain potential study. Neuropsychologia.

